# The Dynamics of EBV Shedding Implicate a Central Role for Epithelial Cells in Amplifying Viral Output

**DOI:** 10.1371/journal.ppat.1000496

**Published:** 2009-07-03

**Authors:** Vey Hadinoto, Michael Shapiro, Chia Chi Sun, David A. Thorley-Lawson

**Affiliations:** Department of Pathology, Tufts University School of Medicine, Boston, Massachusetts, United States of America; Emory University, United States of America

## Abstract

To develop more detailed models of EBV persistence we have studied the dynamics of virus shedding in healthy carriers. We demonstrate that EBV shedding into saliva is continuous and rapid such that the virus level is replaced in ≤2 minutes, the average time that a normal individual swallows. Thus, the mouth is not a reservoir of virus but a conduit through which a continuous flow stream of virus passes in saliva. Consequently, virus is being shed at a much higher rate than previously thought, a level too high to be accounted for by replication in B cells in Waldeyer's ring alone. Virus shedding is relatively stable over short periods (hours-days) but varies through 3.5 to 5.5 logs over longer periods, a degree of variation that also cannot be accounted for solely by replication in B cells. This variation means, contrary to what is generally believed, that the definition of high and low shedder is not so much a function of variation between individuals but within individuals over time. The dynamics of shedding describe a process governing virus production that is occurring independently ≤3 times at any moment. This process grows exponentially and is then randomly terminated. We propose that these dynamics are best explained by a model where single B cells sporadically release virus that infects anywhere from 1 to 5 epithelial cells. This infection spreads at a constant exponential rate and is terminated randomly, resulting in infected plaques of epithelial cells ranging in size from 1 to 10^5^ cells. At any one time there are a very small number (≤3) of plaques. We suggest that the final size of these plaques is a function of the rate of infectious spread within the lymphoepithelium which may be governed by the structural complexity of the tissue but is ultimately limited by the immune response.

## Author Summary

Epstein-Barr virus is a human pathogen associated with several human cancers that nevertheless persists benignly as a latent infection in the majority of adults. EBV persistence is characterized by the presence of latently infected cells in the blood and the shedding of virus into saliva. We present the first systematic quantitative analysis of virus shedding. We show, contrary to what was previously thought, that shedding is continuous and at a high level for all subjects tested. This constant presence of infectious virus may be a crucial risk factor in the development of the EBV-associated tumor nasopharyngeal carcinoma. Unlike infected cells in the blood, which are maintained at very stable levels for years, we show that virus shedding is highly variable such that at any time any individual may be a relatively high or low shedder. We have analyzed these dynamics mathematically and with a simple simulation model. We find that they can be explained by a simple exponential function which we hypothesize is the expansion of 1–3 plaques of epithelial cells.

## Introduction

Epstein-Barr virus is a human herpes virus that has the capacity to transform B lymphocytes in culture into continuously proliferating lymphoblasts [1],[2]. However, it persists in vivo in resting, germinal center derived memory B cells [2],[3] that we refer to as mB_Lat_ [4]. The generally accepted model of viral persistence holds that the virus uses its transforming abilities to activate newly infected resting B cells and then latency associated viral proteins provide surrogate signals to allow the newly activated cells to differentiate through the germinal center into the memory compartment [1]–[3].

EBV is clinically important because of its involvement in several human cancers including Hodgkin's disease, Burkitt's lymphoma, immunoblastic lymphoma in the immunosuppressed and nasopharyngeal and gastric carcinomas [5],[6]. The ability to immortalize B cells and the fact that EBV encodes for several potential oncogenes [7],[8] provides one source of risk for EBV associated tumorigenesis. A second factor in EBV associated cancer susceptibility is that the virus establishes a persistent infection resulting in lifetime exposure of the host to infectious virus and viral gene products. The tissue that is most exposed to infectious virus is the epithelium of the tonsils and nasopharynx since this is where the virus is replicated and shed into saliva for horizontal spread of the infection to new hosts [9],[10]. Long term exposure to infectious virus in this anatomical region may be an important risk factor in the development of the EBV associated cancer nasopharyngeal carcinoma.

Although virus shedding is a hallmark of persistent infection it has not, to our knowledge, been systematically studied in healthy carriers of the virus by sensitive, quantitative PCR techniques. Currently, therefore, there is a dearth of systematic quantitative data on viral shedding that would allow the formulation and testing of models of virus production. Consequently it remains unclear what regulates the levels of virus shedding in saliva. Some previous studies have used a biological assay to detect EBV [10],[11], based on its ability to transform B cells in culture. However, in our hands this assay is unreliable, insensitive and not quantitative. Others have used relatively insensitive and therefore non-quantitative PCR approaches to assess virus shedding particularly during acute infections e.g. [12]–[15]. Perhaps not surprisingly these studies have produced conflicting results. For example one study concluded that virus shedding was correlated with the levels of infected B cells in the blood whereas a second study found no such correlation [11],[13]. Generally however, it appears to be the view in the field that individuals can be classified by relative levels of shedding into high, intermediate and low shedders where virus shedding is sporadic and often undetectable [5],[11]. Since these conclusions are based on non-quantitative and insensitive assays it is difficult to assess and reconcile these conclusions. For example, it is unclear if failure to detect virus is due to its absence or simply a function of the insensitivity of the assays used.

We have previously developed sensitive, quantitative PCR techniques for detecting EBV DNA [4],[16] and EBV gene products [17],[18]. Using these assays we showed for the first time that the levels of virus infected cells in the blood of healthy carriers were individual specific and highly stable over long periods of time (years) [19]. In this study we have set out to apply this same approach to the dynamics of virus shedding into saliva by healthy carriers of the virus and use this data to develop and test potential models of EBV shedding. We find that the overall dynamics strongly implicate the epithelium as a site where virus is amplified prior to shedding.

## Results

### High levels of virus shedding by healthy carriers of EBV

**Figure 1:**
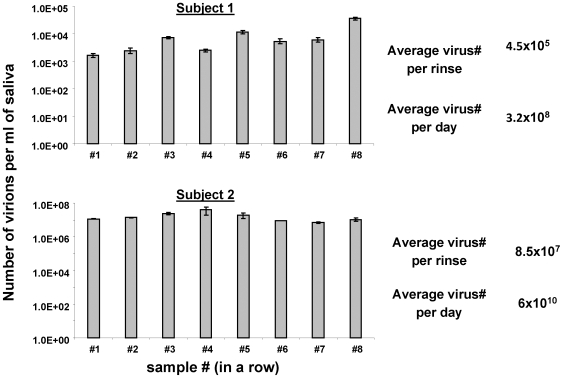
The level of virus in saliva is stable and cannot be eliminated by repeated washing. Two subjects were asked to rinse repeatedly with 5 ml of fluid and the viral genome copy number in the rinses estimated by EBV specific DNA PCR for the W repeat sequence of the viral genome. Note that even after 8 sequential rinses a similar level of virus was detected. The calculation to the right is based on an average time between rinses of approximately 2 minutes and a final volume of saliva rinse of 5 ml.

Traditionally the levels of virus in saliva have been assessed at a single point in time by having subjects gargle with a defined volume of fluid. The amount of virus in the gargle is then measured either with a biological assay [10],[11], based on transformation of B cells in culture or by PCR for the copy number of viral genomes [12]–[15]. However, this type of analysis is static and provides no kinetic information about the rate of virus production. Therefore in our first series of studies we sought to measure the rate of virus production by first rinsing out all of the virus in saliva and then measuring the rate at which the virus returned. To this end volunteers were asked to rinse and gargle sequentially with 5 cc of fluid and the levels of virus measured with a quantitative DNA PCR technique that can detect a single copy of the viral genome. To our surprise sequential rinses failed to deplete the levels of virus in the saliva even when up to 8 rinses in a row were tested (Figure 1 shows the results for two subjects who were at the lower and upper levels respectively of shedding at the time of the experiment). This held true even when the subjects attempted to wash out the virus first with 4 sequential 25 ml rinses and then with 2 more 25 ml rinses. After each attempt a subsequent 5 ml rinse had essentially the same amount of virus as the initial rinse (see Figure S1). We reached the unexpected conclusion therefore that virus is continuously being shed into saliva at such a high rate that the virus removed by rinsing was already replaced by the time we retested the saliva (∼2 minutes). To our knowledge this is the first time that this simple technique of sequential sampling has been performed and leads us to the conclusion that the rate of virus production is much greater than had been previously thought. Included in Figure 1 is the estimated virus shedding per day for the two subjects analyzed which lead to the conclusion that 5×10^5^ and 10^8^ genomes are being produced by Subject 1 and 2 respectively per rinse or 3.2×10^8^ and 6×10^10^ per day.

### Estimated cell number required for viral shedding

The time course of virus production by individual cells in culture has been estimated to be approximately 24 hours from initiation of lytic gene transcription to the release of infectious virus [20]. However, to our knowledge no-one has attempted to accurately estimate the viral burst size. We have done this multiple times for both B cell lines (Akata and B95-8 ) and epithelial cell lines (HONEAkata and AGSBX1). The fraction of cells replicating the virus was assessed by flow cytometry after staining for the viral capsid antigen 48 hours post stimulation. The amount of virus released was measured by performing EBV specific DNA PCR on the cell culture supernatants at 72 hours. The results of this analysis are summarized in Table 1. For the B cell lines the average production was quite similar despite their very different origins or whether or not the virus was chemically induced. Similarly for the two epithelial cell lines. However, production from the epithelial cells (6.2×10^5^genome copies per cell) was about ten fold higher than from the B cells (7.3×10^4^ genome copies per cell)However less than 10% of these genomes appeared to be intact virions based on resistance to DNase digestion. (Table 1).

**Table 1:** Estimated viral burst size for two independent B and epithelial cell lines.

Cell line	Cell type	n	Genome copies/cell	SD	Virions/cell†	SD
Akata+PMA/Iono*	B	5	6.8×10^4^	3.5×10^4^	2.1×10^3^	1.5×10^3^
B95-8+PMA*	B	6	1.0×10^5^	9.2×10^4^	6.3×10^3^	3.4×10^3^
B95-8	B	6	6.2×10^4^	5.7×10^4^	5.9×10^3^	5.7×10^3^
Average	B	15	7.3×10^4^		4.8×10^3^	
AGSBX1+TPA/BA*	Epith.	4	2.6×10^5^	1.0×10^5^	2.3×10^5^	9.7×10^4^
AGSBX1	Epith.	3	1.4×10^5^	2.8×10^4^	1.7×10^5^	6.0×10^4^
Hone1Akata+TPA/BA*	Epith.	4	1.4×10^6^	3.4×10^5^	2.2×10^5^	1.2×10^5^
Hone1Akata	Epith.	3	6.8×10^5^	3.6×10^5^	4.3×10^4^	3.1×10^4^
Average	Epith.	14	6.2×10^5^		1.7×10^4^	

*: Induced by PMA, PMA+Ionomycin or PMA+butyric acid.

n = number of measurements.

For details see Materials and Methods.

**†:** Virion copy number was defined as the portion of viral DNA that was resistant to DNase treatment under conditions where >99% of cell free DNA was digested: 1 U/ul for 30 min at room temperature (see Materials and Methods, Table 3 and Figure S3).

Using a burst size of 7.3×10^4^ genome copies per B cell and assuming a 24 hour lytic cycle we can estimate that the shedding by Subjects 1 and 2 would require viral production from 4.4×10^3^ and 8.2×10^5^ B cells per day, respectively. This is several logs higher than the value we have measured previously for the number of lymphocytes in Waldeyer's ring that complete virus replication which we estimate to be extremely small averaging 0.5 cells per day with an upper bound <100 (Box 1). Therefore, we may conclude that there are insufficient B cells replicating EBV in Waldeyer's ring to account for the levels of virus being shed into the saliva (see also Box 1 and Discussion).

**Box 1. Neither the absolute levels nor the variation over time of virus shedding can be accounted for by B cells alone replicating the virus in Waldeyer's ring**

The frequency of infected B cells in Waldeyer's ring from a large cohort of healthy carriers varies between 1/10^7^ and 1/10^4^ [27].

Assuming 10^10^ B cells in the entire Waldeyer's ring then there are at most 10^6^ infected B cells. We have measured the fraction of infected B cells replicating the virus per day (assuming a 24 hr lytic cycle) to be no more than 0.5%* [26].

Therefore, there are at most ∼5×10^3^ cells replicating the virus.           A.

Alternatively: In a screen of palatine tonsils we detected viral replication in 8 out of 20 subjects [26]. Applying Poisson statistics this suggests that on average there are 0.51 cells per Palatine tonsil or 6.5 per Waldeyer's ring replicating the virus at any time.          B.

However we have shown previously that only 10% at most of cells that initiate viral replication in Waldeyer's ring complete it.

Therefore at most only ∼5×10^2^ B cells complete viral replication per day (from line A) with an average of only 0.65 per day (from line B).

From Table 1 the burst size per B cell = 7.3×10^4^ genome copies.

Therefore, the maximum amount of virus that could be produced by B cells in Waldeyer's ring

 = 5×10^2^×(7.3×10^4^)   = 3.6×10^7^ genome copies per day              C.

And the average per day

 = 0.65×(7.3×10^4^)   = 4.7×10^4^ genome copies per day

From Figure 5 the range of shedding for 8 healthy carriers

 = 5 to 2×10^7^ genomes/ml/rinse

If a rinse takes on average 2 minutes and contains 5 ml then the range of shedding per day for 8 healthy carriers

 = (5 to 2×10^7^ genomes/ml)×5 ml/rinse×30rinses/hour×24 hours

 = 1.8×10^4^ to 7.2×10^10^ genome copies per day                    D.

Comparison of line C and line D reveals that there is a ∼3 log discrepancy between the maximum level of virus shedding that could be accounted for by B cells and the upper limit that we have actually measured.

*N.B. This value is based on estimates from 2 tonsils that had the highest signal for expression of the viral lytic gene BZLF1 therefore it represents the upper limit on the % of infected cells replicating the virus.

### Stability of virus shedding over time

**Figure 2:**
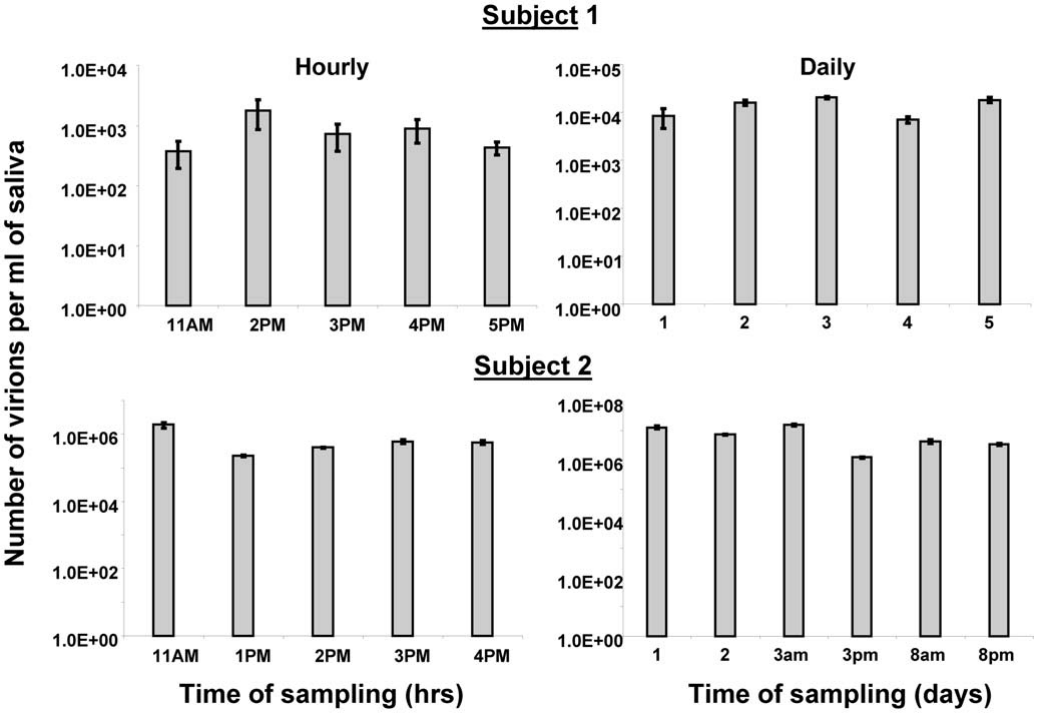
Virus shedding is stable over hours and days. The bars represent the levels of viral DNA found in saliva at the times indicated. For details see Legend to Figure 1. N.B. For the data in the lower right panel single samples were taken on day 1 and 2 but two samples taken on days 3 and 8, one in the morning(am) and one in the evening (pm).

**Figure 3:**
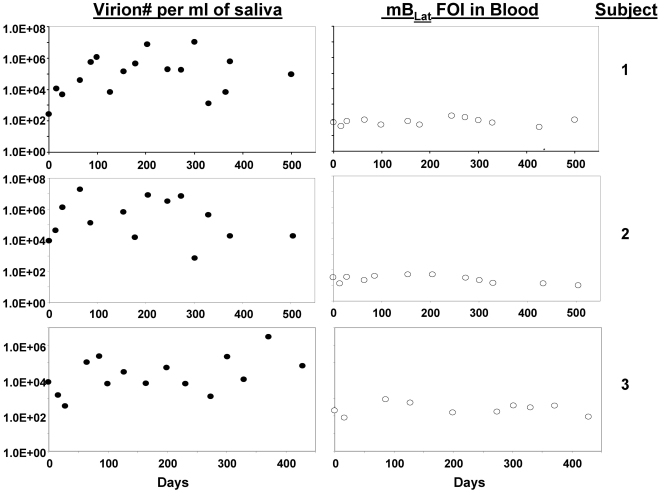
Virus shedding but not levels of infected cells vary by 4 to 5 logs over the course of months/years. The amount of virus shed in saliva and the frequency of latently infected mB_Lat_ in the blood were measured concomitantly in three individuals over the course of 500 days. Note that virus shedding varies over 4 to 5 logs whereas the frequency of mB_Lat_ varies by less than a single log.

Figure 1 demonstrates that virus shedding into saliva was quite stable at least over short time periods. When we measured shedding in the same individuals over hours or days we saw a similar degree of stability (Figure 2). However, when we tracked them for longer periods (up to one and a half years) we saw a strikingly different result (Figure 3) with fluctuations over 4–5 logs. To test if this variation was a consequence of variable “aggressiveness” in the sampling procedure over time we also performed PCR for an internal cellular control gene (bcl-2). When the levels of cellular DNA in saliva were tracked over the course of three months in three individuals the largest variation in recovery we obtained was 40 fold compared to 1.5×10^4^ fold in virus shedding in the same samples (not shown) This indicates that sampling variation was not the source of the variation in virus shedding that we observed. Unlike virus shedding we have shown previously that the level of latently infected memory B cells (mB_Lat_) in the blood of healthy carriers is extremely stable over years [19] and even decades (DTL unpublished observations) in healthy carriers. To confirm that this was true for the subjects and times studied here we measured the levels of mB_Lat_ in their blood at the same times we measured virus shedding. As expected the levels of mB_Lat_ in their blood were stable over the entire time period (Figure 3).

We conclude that although virus shedding within an individual is quite stable over short time periods (hours/days) it is highly variable over the course of months and years. As described above we have previously shown that the number of B cells completing viral replication per day in Waldeyer's ring is small therefore it is also not possible to account for the variation in shedding within an individual simply in terms of variation in the number of B lymphocytes replicating the virus in Waldeyer's ring at any one time (see also Discussion).

### Subjects cannot be distinguished as high, medium or low shedders

**Figure 4:**
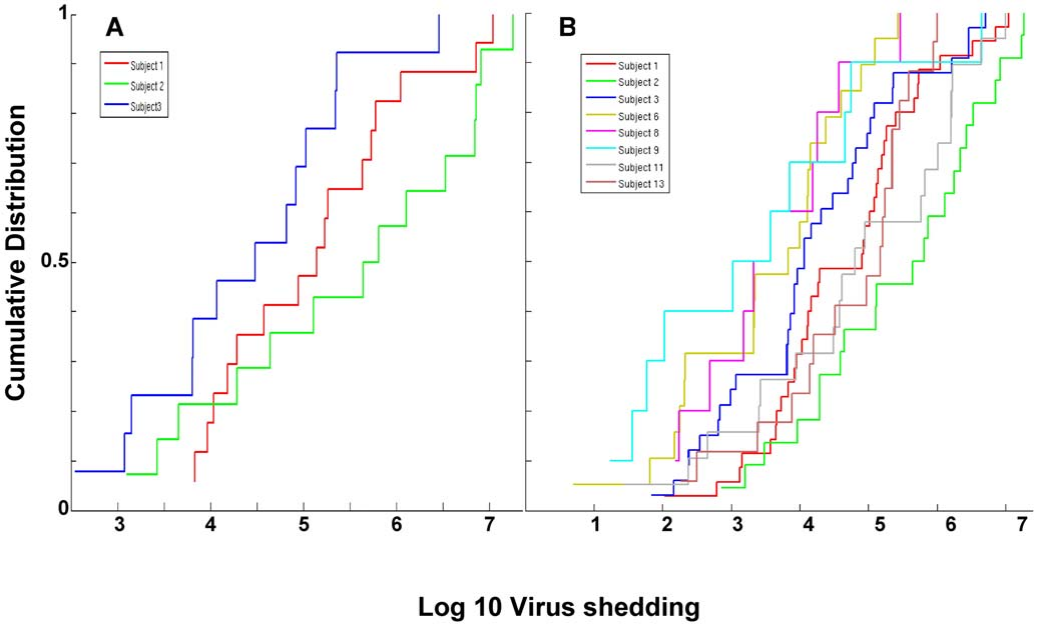
Cumulative distribution function (CDF) for the shedding data. (A) The log_10_ values for the shedding data in Figure 3 are plotted cumulatively from lowest to highest against the normalized number of data points. Each step up represents a data point. Thus the extreme left point on each graph represents the lowest value for which there is only one data point, the next step up represents the second lowest point and so on till the last most extreme right point represents the highest value and therefore includes, cumulatively, all of the data points. (B) Cumulative distribution function (CDF) for all the shedding data summarized in Figure 5A.

**Figure 5:**
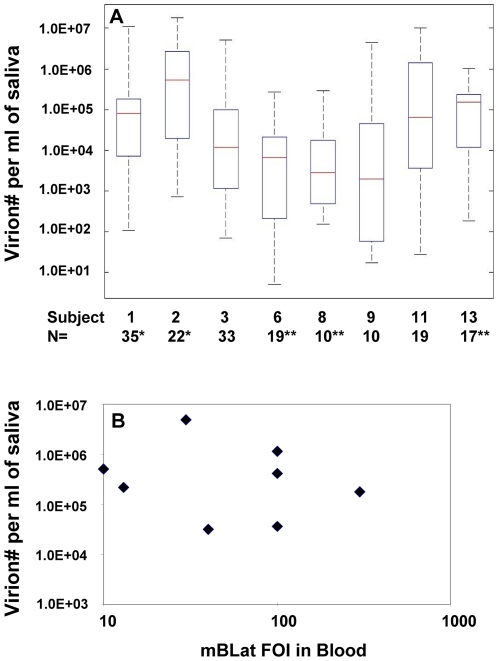
There is no such entity as a consistently high or low shedder of EBV and no correlation between the levels of shedding and the frequency of infected mB_Lat_ in the blood. (A) Shows the range of virus shedding in 8 individuals over months/years. Note that shedding varies between 3.5 and 5.5 logs. Red bars = the median, black box = 25th and 75^th^ percentile, dashed line = the range, N = the number of independent positive measurements. * - the number of asterisks indicates the number of time points when no virus shedding was detected. (B) Shows the mean values for the data shown in A. plotted versus the frequency of infected mBLat (FOI) in the peripheral blood for each subject.

We have analyzed the data from Figure 3 to see if it contained an underlying structure that might shed light on the dynamics and mechanism of virus shedding. Most revealing was the cumulative distribution function (CDF). The CDF (http://en.wikipedia.org/wiki/Cumulative_distribution_function) describes the probability distribution of a real-valued random variable X, in this case the log value of virus shedding. The CDF of a given value of X is the proportion of the measurements whose value is equal to or less than X. As the value of X increases, the CDF of X increases from 0 to 1. So for example the median for a set of measurements has a CDF of 0.5. The CDF for the three subjects in Figure 3 are presented in Figure 4A. Here the log values for virus shedding are plotted cumulatively from lowest to highest and thus appear as steps, and the number of data points are normalized by setting the total to equal 1. Strikingly the distributions were essentially linear for all three data sets. To see if this was a coincidence unique to this particular experiment or a general property of virus shedding we analyzed a large data set collected from 8 different subjects over an extended period of time (up to two years) which also included a much larger number of data points for subjects 1–3 (essentially additional points for which we did not have matching blood level measurements). A summary of all this data, which includes a total of 165 positive data points and 8 time points where no virus shedding was detected, is presented in box plots in Figure 5A and in Table S1. The time points where no virus shedding was detected are not included in the box plots but are indicated by the asterisks in Figure 5A. We are confident that there was no viral DNA present in the saliva samples at these times because our PCR technique can reproducibly detect a single copy of the viral genome and positive detection of a control cellular sequence (bcl-2) confirmed the sampling process was normal.

**Figure 6:**
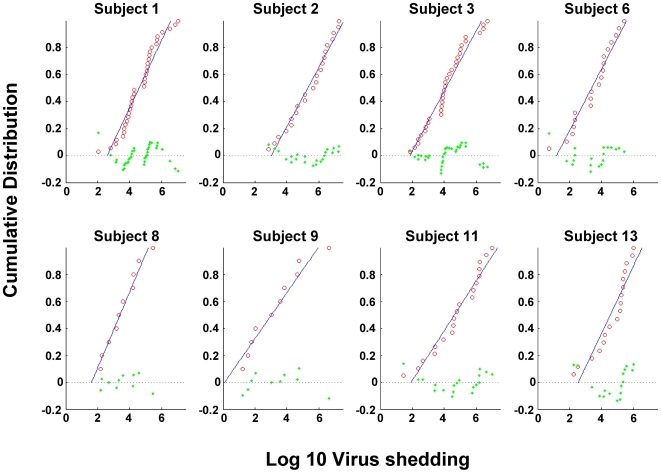
Linear regression analysis of the data in Figure 4B. The CDFs for the 8 subjects are plotted individually as data points rather than as steps with the best fit straight line from linear regression analysis. Slopes and adjusted R^2^ values are given in Table 2. Blue line = the best fit straight line from the linear regression analysis. Red circles = the data points; green circles = the residuals i.e. the difference between each data point and the value given by the line.

From Figure 5A it is apparent that a large range of variation is a ubiquitous feature of virus shedding with most subjects demonstrating around a 4 log range, the largest being 5.5 logs (subject #9) and the smallest 3.3 logs (subject #8). Table S1 shows the mean, median and standard error of the mean (SEM) for the measurements on all 8 subjects Although the mean and median values only varied by about two orders of magnitude between the 8 subjects, the SEM within each individual ranged over 4–6 orders of magnitude indicating the extent to which the range of virus shedding overlapped between individuals and that the shedding profiles are not statistically different between different individuals. Most revealing though was the CDF analysis of this data shown in Figure 4B which confirmed the original result with the limited data set from subjects 1–3. For all 8 subjects, a linear relationship was observed between the log value of the shedding and its cumulative probability of occurring. Put another way the probability of (log)shedding was equal for all levels over a several log range. This is the expected result when a simple exponential process, such as growth or infectious spread, is sampled randomly over time. In Figure 6 we show the best fit lines to the data from all 8 Subjects (from Figure 4B) and in Table 2 we describe the properties of these lines including the slopes and the results of linear regression analysis (adjusted R^2^). The R^2^ value for all 8 subjects was extremely high (≥0.88) indicating that the data closely approximates a linear relationship. In addition it is apparent that the slopes of the lines are very similar for all 8 subjects although the lines were spread over a wide range encompassing >2 logs (compare Subject 2 and 9 in Figure 4B and the x intercepts for all 8 subjects in Figure 6).

**Table 2:** Slope and R^2^ values for the best fit lines shown in Figure 6.

Subject	Slope	Adj R^2^
1	0.25	0.94
2	0.22	0.98
3	0.22	0.96
6	0.23	0.94
8	0.28	0.97
9	0.17	0.94
11	0.18	0.95
13	0.25	0.88

These results provide several important conclusions. Contrary to what has been claimed previously [11], there are no distinct high, intermediate or low virus shedders. So although on average for example subject #9 is low, subject #3 medium and subject #2 high, multiple measurements over extended time show these assignments to be relatively meaningless. Rather at any one time an individual might be high, low or intermediate. Furthermore the underlying process behind the variation in shedding is a simple exponential that is either arrested randomly, and then held stable, or observed at random times. This exponential is initiated over a range of shedding at levels that are subject specific (lines are displaced over >2 logs in the CDF plots) but whose subsequent growth rate is subject independent (slopes are similar in the CDF plots). We suggest that these events are the exponential expansion of infected epithelial cell plaques (see Discussion).

### Simulation of the exponential function shows the number of independent events is very small

**Figure 7:**
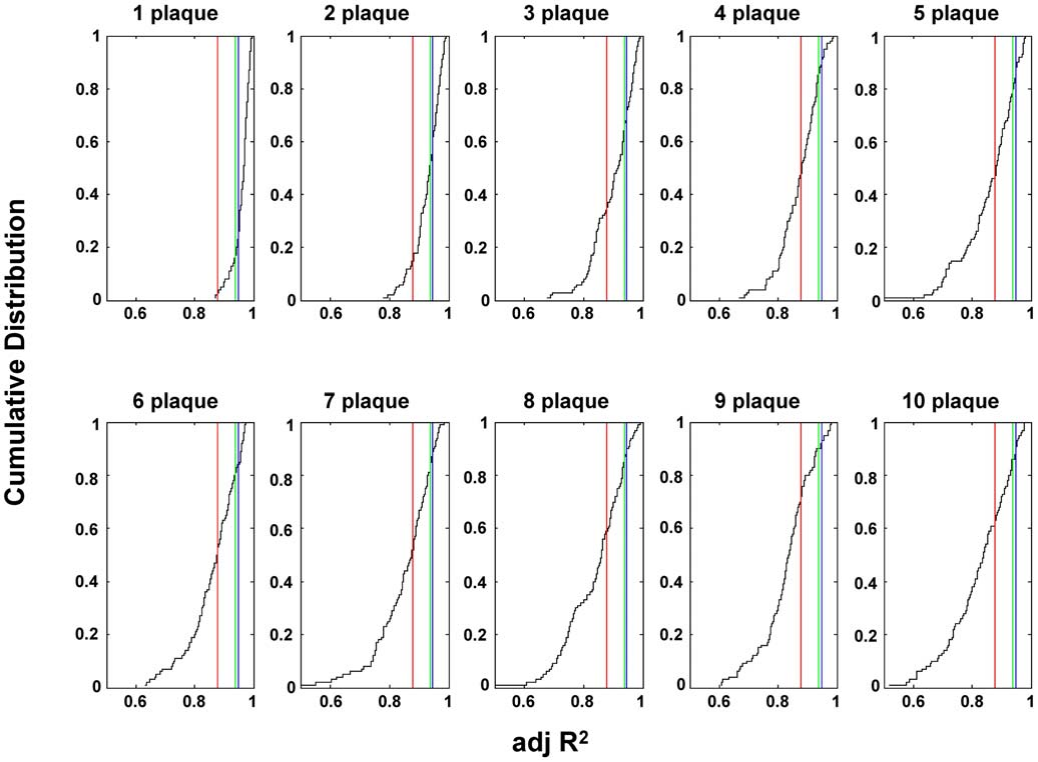
Adjusted R^2^ values for simulations of plaque. A simulation was performed where infectious plaques were initiated randomly, allowed to grow exponentially and randomly sampled 25 times (see Text S1 and Figure S2). This data was then plotted as a CDF and the adjusted R^2^ estimated for the best fit straight line to the data. This process was repeated 100 times for each of the plaque numbers indicated and the resulting R^2^ values plotted as a CDF. The red line indicates the lowest (0.88), the green line the 7^th^ lowest (0.94) and the blue line the median R^2^ for our 8 subjects (see Table 2).

A straight line relationship between the log of shedding and the cumulative number of observations (the CDF) implies that the number of these putative plaques must be very small otherwise the shedding from the largest plaques would always dominate and the plot would become non-linear. This is demonstrated in Figure 6 and Figure S2 where we have performed a simulation of this process for various numbers of plaques (For details of the simulation see Text S1 – “Empirical CDF of shedding suggests there are at most 3 plaques at any one time.”). In this simulation various numbers of plaques are seeded randomly, allowed to grow exponentially and then sampled at 20 random points in time. This produces a simulated CDF of log shedding from which an adjusted R^2^ can be calculated. As the number of plaques increases the plots tend to become steeper and increasingly skewed away from linear. We next used the simulation to ask: what is the probability of obtaining our actual data set with different numbers of plaques? To achieve this we simply repeated the simulations shown in Figure S2 100 times for each plaque number, performed a linear regression analysis to calculate the adjusted R^2^ for each run and then plotted these values as a CDF for the adjusted R^2′^s (Figure 7). We can then ask: what is the probability of obtaining the adjusted R^2^ values we have observed for our 8 subjects with simulations involving different numbers of plaques. We have used three different criteria. First, what is the probability of obtaining an adjusted R^2^ value 7 out of 8 times in a row that is higher than the lowest value we have observed with our 8 subjects? Second what is the probability of obtaining 8 values with a median value equal to or higher than the median value for our 8 subjects? Third, what is the probability of obtaining 8 values as high as or higher than the lowest value we have observed with our subjects. This calculation revealed p values of <0.05 for ≥2 plaques, ≥3 plaques and ≥4 plaques for the three methods respectively. Taken together these analyses suggest that the shedding of EBV in healthy carriers is the result of a very small number, ≤3, of these putative epithelial plaques.

We can also derive an estimate of the average plaque number in a completely different and independent way since we have observed that virus was not shed on 8 occasions out of the total 173 measurements we have taken. From simple Poisson statistics we can calculate that if we find no evidence of infectious plaques 8/173 times then on average there must be ∼3 such plaques, a value which is in very close agreement with the prediction (≤3) of the CDF analysis.

In conclusion it appears that EBV shedding is the consequence of an exponential process that is either terminating or being observed randomly and that there are ≤3 of these events occurring at any given time.

### Levels of shed virus do not correlate with the levels of mB_Lat_ in the blood within or between individuals

As noted above the levels of mB_Lat_ in the blood of healthy carriers of EBV is stable over long periods of time ([19] and Figure 3) and we have now shown conversely, that virus shedding is extremely variable over similar time spans. Therefore there is no correlation within individuals between virus shedding and levels of infected cells. In Figure 5B we have plotted the mean values from Table S1 versus the frequency of mB_Lat_ in the circulation for all subjects studied and it is apparent that there is also no correlation between the levels of mB_Lat_ in the blood and the amount of virus shed into saliva between individuals. (The same result holds true if the median is plotted, not shown).

**Figure 8:**
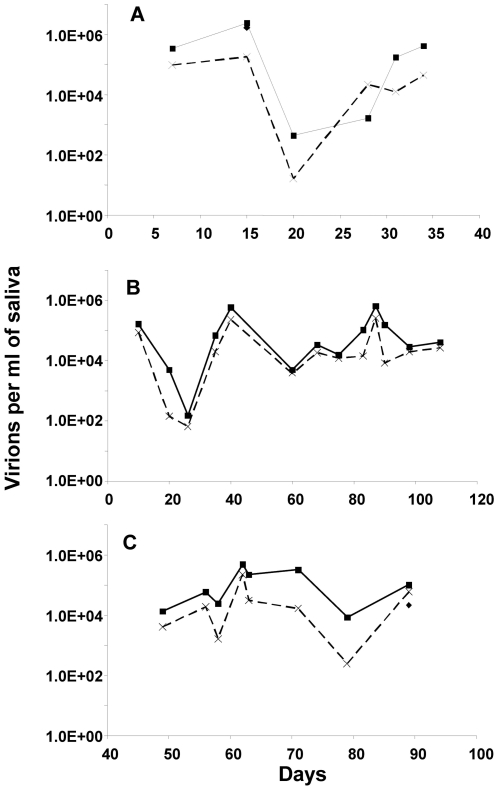
The variation in virus shedding into saliva over time is consistently seen in clarified and DNase treated preparations. Solid line = Saliva was pretreated with DNase to remove unencapsidated viral DNA. Broken line = Saliva was clarified by centrifugation and then treated with DNase. Data is shown for three different subjects (A–C).

One possible explanation for the lack of correlation between shed virus and the levels of mB_Lat_ in the blood is that PCR does not distinguish intact virions from non-infectious viral DNA. To evaluate this we assessed the DNase sensitivity of viral DNA in saliva (see Figure S3) for an example of such an experiment). We found that most of it was resistant; consistent with being in intact virions, whether we tested raw saliva or the supernatant after clarification by centrifugation (Table 3). This was much higher than for the 2 cell lines we tested Akata and B95-8 (only 10–20% resistant) suggesting that virions shed into saliva are more efficiently packaged than in the cell lines. When we tracked the levels of DNase resistant material in raw or clarified saliva we again saw a 4 to 5 log variation over extended periods of time (Figure 8) and no correlation with the level of mB_Lat_ (Figure S4).

**Table 3:** Most saliva derived virions are DNase resistant unlike those derived from cell lines.

Virus Source	% DNase resistant	
	Ave.*	SD
B95-8	17	4.5
Akata	4	3.5
Saliva	62	12
Spun Saliva	59	5.5

*: The average was derived from 3 independent measurements for Akata and B95-8 cell lines and from measurements for 3 individuals each measured independently twice. See Figure S3 for an example from one subject.

The other possible explanation for our failure to find a correlation between shed virus and mB_Lat_ was that PCR measures all virions irrespective of their infectivity and it has been suggested that a large fraction of virions in saliva are bound by neutralizing antibody [21]. To test this possibility we assayed the amount of viral DNA that would bind specifically (as judged by the failure to bind the T cell line Jurkat and the ability to be blocked with anti-gp350 neutralizing antibody 72A1 – not shown) to a B cell line. As shown in Table 4 in two separate experiments despite repeated absorptions (up to 6 times) only <5% of the viral DNA in saliva could bind the B cell line Akata. However, when we tested the amount of Akata binding activity in saliva over an extended time period we again found that the levels varied by approximately 4 to 5 logs and that there was no correlation with the levels of mB_Lat_ in the peripheral blood (Figure S5).

**Table 4:** Most viral DNA in saliva does not bind to B or epithelial cells.

Absorption	Low # (5×10^3^) virus added		High # (5×10^5^) virus added	
	# virions bound		# virions bound	
	Akata	AGS	Akata	AGS
#1	624	833	1.6×10^5^	1.4×10^5^
#2	142	76	2.5×10^4^	1.7×10^4^
#3	64	4	6.0×10^3^	3.6×10^3^
#4	13	10	4.3×10^3^	2.0×10^3^
#5	24	0	1.4×10^3^	1.0×10^3^
#6	0	0	8.5×10^2^	9.8×10^2^
% unbound	98	98	96	97

A low and high amount of saliva virus was subjected to repeated absorption with the B cell line Akata and the epithelial line AGS. The amount of bound virus was assessed after each absorption. Note that >95% of the virus remained unbound.

## Discussion

**Figure 9:**
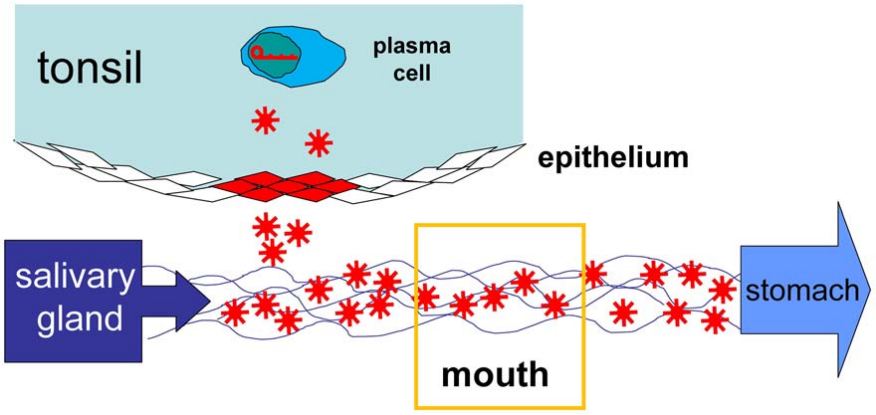
Schematic representation of the proposed model from this study. EBV infected mB_Lat_ that return to the Waldeyer's ring (tonsils and adenoids) undergo plasma cell differentiation and initiate viral replication. In a typical healthy carrier the number of such cells at any one time is ≤50 and therefore insufficient to account for either the absolute level or the variation in shedding that we have observed over time. Virus released from the plasma cells infects the epithelium of Waldeyer's ring where it forms a plaque containing anywhere from 1 to 10^5^ infected epithelial cells thus amplifying the amount of shed virus. Variability in the final size and stability of these plaques accounts for the high levels and variability of virus shedding and is probably a function of the initial seed of infected epithelial cells, the epithelial structure, and the immune response. Virus from the plaque is continuously shed into the saliva for several days providing a continuous stream of virions in the saliva that is replaced≤every 1 to 2 minutes.

In this paper we have described detailed kinetics of EBV shedding in the saliva of healthy carriers of the virus. We have employed a sensitive and quantitative DNA PCR assay that can reliably detect a single copy of the viral genome. Using this assay we show for the first time that EBV positive individuals continuously shed EBV such that the saliva reservoir is completely replaced in ∼2 minutes. This is consistent with studies showing that a normal individual swallows on average once every 2 minutes (www.uiowa.edu/~c003236/indexS1L1a.html). Thus the mouth is not a reservoir of virus but a conduit through which a continuous flow stream of virus passes in saliva (Figure 9). Over the course of several months to a year the level of shedding by a given individual varies by 3.5 to 5.5 logs. Thus contrary to what is currently thought [11] there is no clear distinction between low, intermediate or high shedders of EBV. In fact shedding changes so much over time that most of the variation between high and low occurs within an individual not between individuals. If a cohort of individuals are only measured over the course of a few days some will be low, some intermediate and some high, but the classifications will change over time. For example in Figure 3 Subject 1 might be classified as high (10^6^ to 10^8^ per ml) on day 300 or low (10^2^ to 10^4^ per ml) on day 325 and intermediate (10^4^ to 10^6^ per ml) on day 500 whereas Subject 2 would be high on day 50, low on day 300 and intermediate on day 325. This point is confirmed by the CDF analysis which demonstrates that an individual has an equal probability of shedding at any level (log values) from high to low.

Previous studies using either a biological assay [22],[23] or PCR [24],[25] to detect the virus have reported that 10 to 70 percent of EBV positive individuals shed detectable virus. In this study we took 173 measurements over time on 8 different EBV positive individuals and consistently found all 8 to be positive with only 8/173 negative readings. Unlike previous studies we can be confident that there was no viral DNA present in the saliva samples at these times because our PCR technique can reproducibly detect a single copy of the viral genome. Therefore, we did not detect an EBV positive individual who was consistently a non-shedder. The much higher rate of success we obtained no doubt reflects that the assays used before were considerably less sensitive. We observed similar discrepancies previously when assessing the prevalence of EBV in peripheral blood B cells. Earlier reports had suggested a wide range of detection based on PCR or a biological assay. However, application of our DNA PCR assay revealed that 100% of EBV seropositive individuals had infected cells in their blood 100% of the time and indeed the levels were very stable and specific to each individual [19]. These studies on viral loads in saliva and blood B cells demonstrate that performing precise, sensitive and systematic quantitative studies can provide consistent and novel insights into EBV biology.

Surprisingly the main conclusions of our studies on viral shedding point to the potential importance of epithelial cells in EBV replication and shedding. This is because neither the absolute levels nor the variation over time of the virus shedding we have observed can be accounted for by B cells alone replicating the virus in Waldeyer's ring [26]. In Box 1 we have attempted to estimate the amount of virus being shed from Waldeyer's ring by B cells. This calculation is based on the number of infected B cells in Waldeyer's ring from measurements on a large cohort of normal subjects [27], our previously published estimates of the fraction of infected B cells that completes viral replication at any time in Waldeyer's ring [26] and the viral burst size as estimated in the current study. From this calculation we can see that there is ∼3 log discrepancy between the absolute maximum amount of virus that could be produced by B cells in Waldeyer's ring and the actual levels we have measured in saliva. Therefore it is highly unlikely that B cells alone can account for the levels of virus shedding we have observed. Besides the absolute levels of virus shedding, the range (3.5 to 5.5 logs) we have observed over time in any given healthy carrier is also too large to be accounted for by B cells alone replicating the virus since we estimated previously that at most ∼100 B cells complete EBV replication in Waldeyer's ring at any one time ([26] and Box 1). This could only yield at best ∼2 logs of variation, again implicating a second site of EBV replication. What/where could this cell type be? Recently the possible role of epithelial cell infection in EBV biology has again been raised. Infected epithelial cells have been detected in cultures of primary tonsil epithelium [28], EBV has been shown to have a cluster of virion glycoproteins (gH and gL) that mediate epithelial cell infection [29],[30] and EBV is found in the epithelial cells of nasopharyngeal carcinoma [31] (where the virus is latent) and oral hairy leukoplakia [32] (where the virus is lytic). Furthermore, the virus in saliva is high in the glycoprotein gp42. This glycoprotein binds to MHC Class II and is usually depleted in virions that emerge from B cells suggesting that the virus in saliva originates from a non-B cell [33]. This is consistent with a model whereby virus released from B cells infects the tonsil epithelium where it replicates and becomes amplified prior to shedding into saliva. To investigate this possibility we have estimated the potential amount of virus produced from epithelial cells in Waldeyer's ring (Box 2). This calculation was based on published values for the surface area of epithelial cells in Waldeyer's ring [34], our estimate of the density of an epithelial cell monolayer [28], our published estimates of the frequency of epithelial cells replicating EBV in cultures of primary tonsil epithelium [28] and the viral burst size of epithelial cells as estimated in this study (Table 1). From this calculation it is apparent that epithelial cell infection could account for the levels of shedding we have seen.

**Box 2. Epithelial cell infection can account for the observed level of virus shedding**

The surface area, including the crypts, of a palatine tonsil is reported to be 340 cm^2^ [34].

A palatine tonsil constitutes approximately one twelfth of Waldeyer's ring [34].

Therefore, the total epithelial cell surface area of Waldeyer's ring is ∼4,080 cm^2^.

From monolayer cultures of primary tonsil epithelium [28] we estimate that 1 cm^2^ of confluent epithelium contains ∼2×10^5^ epithelial cells.

Therefore, 4,080 cm^2^ contains ∼10^9^ epithelial cells.

Since in actual tonsils the epithelium is multilayered the total number of epithelial cells in Waldeyer's ring approximates ∼10^10^.

In primary cultures of tonsil epithelium we have seen ∼0.01% of the cells to be lytically infected with EBV [28].

Therefore, ∼10^6^ epithelial cells may be lytic in the entire Waldeyer's ring of these subjects.

Given an average burst size for epithelial cells of 6.2×10^5^ and assuming a burst time of 24 hours:

Typical virion production from the epithelium could be ≈6.2×10^11^ genomes per day.    E.

Comparing line E with line D in Box 1 it is apparent that the epithelium could account for the levels of virus shedding we have measured.

The CDF analysis provides considerable insight into how epithelial infection could account for the shedding we have observed. We have seen that the CDF for the log of shedding is close to linear for each subject. A linear CDF occurs when a process grows exponentially with respect to time over a fixed life span and is either sampled at random or arrested and held constant at random times. We can discount the first hypothesis (sampling) because virus shedding is stable over short periods of time and is therefore not simply an exponentially growing function. Rather it appears to be an exponentially growing function that is stopped at random, stays stable for several hours/days and then dissipates. Since virus shedding cannot be accounted for by B cells we propose that this function is the growth of infected epithelial plaques that are seeded by virus released from B cells and that these plaques become arrested in their spread randomly over time. This would be the EBV equivalent of HSV reactivating in a ganglia traveling to the skin and infecting the surrounding fibroblasts leading to the production of a herpes lesion. Assuming a burst size of 6.2×10^5^ for epithelial cells (Table 1) the range of virus shedding/ml saliva rinse we have observed in Figure 5A and Table S1 (5−2×10^7^ genomes yields [5−2.10^7^]×5 ml/rinse×30 rinses/hour×24 hrs = 1.8×10^4^ to 7.2×10^10^ genomes per day) would derive from <1 to 10^5^ epithelial cells releasing virus per day. This implies that the plaques range in size anywhere from 1 cell (which could simply be the original seeding B cell) up to 10^5^ epithelial cells.

As demonstrated in the results section the linear nature of the CDF for our subjects constrains the number of these plaques to be ≤3 at any time. However if this is true why do we not see periods of simple exponential growth in our measurements? When we perform measurements at ever shorter time periods we eventually encounter stability not exponential growth. This observation puts two constraints on the model, The first is that the number of plaques is usually >1 such that a plaque in its stable state is usually masking part or all of the growth phase of a new plaque(s) and second that the maximum time of growth for the plaque must be less than the length of time they are stable which we have seen is up to 7 days (For a detailed discussion of this issue see Text S2 – “Why we have not observed the growth phase.”. This places the maximum life time for a plaque at 14 days: at most 7 days of expansion followed by <7 days of stability.

The two obvious candidates for the exponential function predicted by the CDF are growth of infected epithelial cells or infectious spread of virus. If growth were responsible it assumes a model where new infection produces latently infected epithelial cells that continue to proliferate and generate cells producing virus at the same time until some event, presumably the immune response halts the growth. Latent infection of tonsil epithelium has been documented [28] and this type of scenario was proposed many years ago based on analogy to the HPV system [35] where the differentiation of latently infected cells triggers viral replication. In support of this idea, differentiation dependent replication of EBV has been documented in both epithelial [36] and B cells [26]. However, we do not favor this model because it would take two to three weeks to achieve the maximal plaques size of 10^5^ cells (assuming the cells divide once every 24 hours). As we have seen the data constrains the growth phase to be no more than a week and in addition it seems highly unlikely that such a structure could evade the immune response for such a long period of time.

The alternative hypothesis is that the plaques simply grow by infectious spread. Precedence for this idea comes from in vitro infection of polarized tonsil epithelium which has been shown to readily generate plaques of cells replicating the virus [37],[38] and in vivo from the pathological entity oral hairy leukoplakia which arises in the immunosuppressed and essentially consists of very large plaques of epithelial cells replicating EBV [32]. In this model a single epithelial cell releasing virions sufficient to infect on average ∼10 or more new cells after each round would reach the maximum plaque size in less than a week. However, there is a geometric constraint which must be considered. The tonsil surface exhibits complex geometry but can be thought of effectively as a large 2-dimensional surface. If infection can only take place between neighboring epithelial cells, then infection is confined to a growing 2-dimensional disc. The radius of this disc depends on the number of rounds of neighbor to neighbor infections, i.e. the length of time the infection has been spreading. Since the area of a disc grows with the square of its radius, then the number of infected cells should only grow in proportion to the time squared. This means that strict neighbor-to-neighbor contact infection will not support exponential growth. To maintain exponential expansion virus would have to spread to targets beyond those in proximal contact but, this virus would normally be removed by neutralizing antibody. However, Tugizov et al have shown the in vitro development of epithelial plaques through the cell to cell spread of infectious virus via a mechanism involving tightly connected lateral membranes, which is time dependent [37] and resistant to neutralizing antibody [38]. In addition they have shown in vitro that EBV can rapidly spread in epithelium by transcellular transcytosis potentially allowing the virus to spread rapidly beyond the adjacent cells allowing exponential expansion of the plaque (Sharof Tugizov personal communication).

A further result from the CDF analysis is that the slopes are similar but the range of shedding is different between subjects. This means that the exponential expansion of the plaques is being initiated at a different size for each subject but once started is constant between subjects. This fits nicely with the idea that very small numbers of B cells are releasing virus that initiates plaque formation and the number of epithelial cells that initially become infected is dependent on the particular subject. This variation could be introduced simply based for example on the efficacy of the neutralizing antibody each subject produces. A soon as epithelial cell infection is achieved the growth of the resulting plaque becomes subject independent again consistent with the results of Tugizov et al that epithelial infection is not affected by neutralizing antibody. If we assume that the lowest value for each subject approximates the initial size of the plaques then we can estimate (assuming an epithelial cell burst rate of 6.2×10^5^ per cell per day) that the initial infection ranges between ∼0 and 5 epithelial cells. We have estimated above that the plaques grow for a maximum of one week and then survive stably for maximally another week and that there are on average about 3 plaques. If each plaque is seeded by a single B cell releasing virus then on average only about 3 such B cells are generated in the Waldeyer's ring every 1 to 2 weeks or 1 cell every 2 to 5 days. Although this seems extremely low it is consistent with our previous findings ([26] and Box 1). As described in Box 1 a screen of tonsils for expression of the immediate early lytic gene BZLF1 suggested that on average around 1 B cell every two days completes the lytic cycle (see Box 1 for the calculation) a result in remarkable concordance with the estimate from the CDF analysis.

Another insight from the CDF analysis is that the exponential expansion is arrested at random time intervals to generate the simple linear step ladders observed in Figure 4. Two explanations seem possible/likely. The first is that the heterogeneous structure of the epithelium imposes a random sized barrier on the expansion. This barrier could take the form of cells that were not infectable by EBV or otherwise prevented the virus from spreading. However this would require the epithelial cells to continue shedding relatively stable quantities of virus for several hours/days. Continuous virus production for several days without dying has not been documented for EBV in vitro however, we have virtually no information about EBV replication in vivo and such behavior has been described for another herpesvirus CMV [39],[40].

The other process that could interrupt exponential expansion randomly over time could be the cytotoxic T cell response. It has been shown that tonsils contain large numbers of EBV specific CTL [14]. Often >1% of CD8 T cells in the tonsil are specific for EBV lytic antigens. That study suggested ∼10% of these CTL were activated at any given time indicating an ongoing immune response. The random log size distribution of the plaques could then simply reflect the random probability that a CTL will encounter the exponentially growing plaque over time and destroy it. This, at least in part, answers the question of why the virus is able to continuously replicate in Waldeyer's ring with impunity despite a persistent ongoing CTL response. A further contributing factor could be that the virus is known to posses immune evasion mechanisms that could delay or help evade the CTL response. These include an IL-10 homologue that suppresses the CTL response and gene products that interfere with antigen presentation (reviewed in [41]). Presumably if small numbers of plaques are regularly being seeded from B cells these evasion mechanisms must be effective enough to allow the plaques to grow and survive for several days. Indeed the relatively stable short term shedding we have observed may simply reflect an extended period of time where the CTL response is only able to contain the spread of the plaque, it taking several days to finally eliminate it.

If we are predicting that EBV is shed via epithelial plaques that range in size up to 10^5^ cells why have they not been observed in immunohistochemical studies of tonsils? The answer almost certainly relies on chance since our analysis predicts that plaques in the range of 10^4^ to 10^5^ cells are only present ∼10% of the time in a random sampling of subjects. Furthermore, these cells are likely contained within a single plaque in the entire Waldeyer's ring. Therefore our results predict that it would take exhaustive and comprehensive analysis of multiple tonsils to have a chance of seeing an infected plaque of epithelial cells.

In summary, the dynamics of EBV shedding seem to favor a model (Figure 9) where single B cells sporadically initiate EBV replication in Waldeyer's ring. The resultant virus seeds a local infection of epithelial cells that grows exponentially, for no more than 7 days, into an infected plaque. Plaque creation occurs at a rate such that there are 1 to 3 plaques of lytic epithelial cell infection at any one time. The size of these plaques depends on the initial number of epithelial cells infected and the ability of the infection to spread and grow. The size is ultimately bounded randomly either by the structural constraints of the epithelium and/or the efficacy of the immune response. Once the plaque has reached its mature size it produces, for a brief time (up to 7 days), a constant rate of cells that initiate lytic replication probably representing a balance between infectious spread and immune containment. This continues for a few days until the plaque ceases production presumably due to destruction by the cellular immune response.

Our results also relate to conclusions drawn previously by other groups. A long standing claim in the EBV field is that the level of infected cells in the blood correlates with the level of virus shedding in saliva [11]. We failed to confirm this finding both between and within individuals. Furthermore this discrepancy cannot be accounted for by the fact that we have used PCR to detect the virus whereas the previous study used a biological assay. We found no correlation whether we measured total shed genomes, DNase resistant genomes (virions) or viral binding. During the course of these studies we observed that, unlike virus produced by cell lines, the majority of saliva virus is encapsidated (10% versus 60%, respectively) but most of it does not bind to cells (<5%) consistent with previous observations that most of the virus may be coated with neutralizing antibodies [21].

In conclusion, detailed studies of virus shedding indicate that healthy carriers of EBV consistently shed large amounts of the virus into saliva. Furthermore the dynamics of shedding suggest a crucial role for epithelial cells in amplifying the amount of shed virus. However in healthy carriers, although most of the shed virus is intact, very little of it is infectious.

## Materials and Methods

### Ethics statement

This study was conducted according to the principles expressed in the Declaration of Helsinki. The study was approved by the Institutional Review Board of Tufts Medical School and Tufts Medical Center (IRB study #731). All subjects provided written informed consent for the collection of samples and subsequent analysis.

### Cells and cell lines

Whole blood samples were obtained from health volunteers. PBMCs were isolated by Ficoll Hypaque centrifugation and B cells were purified by negative selection with a StemSep column following the manufactures instructions. The efficiency of purification was checked by staining for CD20 and FACS analysis. B cells were always 90–96% pure. Memory B cells were isolated from purified B cells using a MoFlo FACS sorter, based on staining with anti-CD27-FITC (memory B cell marker) and anti-CD20-PE (pan B cell marker).

The EBV positive lymphoblastoid line IB4 (gift from Dr Elliot Kieff) was used as a positive control and the murine CB59 T cell line (gift from Dr Miguel Stadecker) as negative control for EBV DNA PCR. The EBV negative Burkitt lymphoma line Akata and the gastric carcinoma cell line AGS (gifts from Dr Lindsey Hutt-Fletcher) were used as target cells for binding assays. The EBV positive B95-8 marmoset B cell (gift of Dr Elliot Kieff), Akata 2A8.1 (gift of Dr. Jeff Sample), AGSBX1 gastric carcinoma (gift from Dr Lindsey Hutt-Fletcher) and Hone1Akata nasopharyngeal carcinoma (gift from Dr Ron Glaser) cell lines were used for measuring the viral burst size.

### Real time PCR

EBV genomes were detected by a Real time *Taq*man DNA PCR assay for the W repeat region as described previously [4]. This assay can detect a single copy of the EBV genome in a background of 10^6^ EBV-negative cells and a single virion in 0.1 ml of saliva (not shown). PCR was performed in a volume of 25 µl on the ABI Prism 5700 (Perkin Elmer) or MyIQ System (BioRad). Thermal cycling was initiated with a denaturation step at 95°C for 3 min, and continued with 50 cycles of amplification at 95°C for 15 seconds and 60°C for 1 minute. For each reaction, 5 µl of DNA template was added to a master mix containing 12.5 µl of Universal *TaqMan* Master Mix (Applied Biosystems or BioRad) and 2.5 µl each of the forward primer, reverse primer and flurogenic probe. The primers used were AGTGGGCTTGTTTGTGACTTCA (forward) and GGACTCCTGGCGCTCTGAT (reverse) and the flurogenic probe was 6-FAM-TTACGTAAGCCAGACAGCAGCCAATTGTC-TAMRA.

To control for variation in sampling over time, the levels of viral DNA in saliva samples were normalized to the levels of endogenous cellular DNA based on detection of the bcl-2 gene. Bcl-2 Sybr Green Real time PCR was performed in a volume of 25 µl. For each reaction, 5 µl of DNA template from shedding assay was added to a master mix containing 12.5 µl of Sybr Green Master Mix (BioRad) and 2.5 µl each of the forward, reverse primers and water. The primers used were CTTTAGAGAGTTGCTTTACGTG (forward) and TCCATATTCATCACTTTGACAA (reverse). Thermal cycling was initiated with a denaturation step at 95°C for 3 minutes and continued with 45 cycles of amplification at 95°C for 15 seconds, 55°C for 15 seconds, 72°C for 30 seconds, and 83°C for 10 seconds. This was followed by melting curve analysis at 95°C for 1 minute, 55°C for 1 minute, and 80 cycles of 55°C for 30 seconds.

To assess the efficiency of DNase treatment samples were assayed for the presence of cellular DNA by Real time Sybr Green PCR for the β-actin gene. PCR was performed in a volume of 25 µl. For each reaction, 5 µl of DNA template from saliva samples was added to a master mix containing 12.5 µl of Sybr Green Master Mix (BioRad) and 2.5 µl each of the forward, reverse primers and water. The primers used were GCGGGAAATCGTGCGTGACATT and GATGGAGTTGAAGGTAGTTTCGTG. Thermal cycling was initiated with a denaturation step at 95°C for 3 minutes and continued with 40 cycles of amplification at 95°C for 15 seconds, 60°C for 20 seconds, and 72°C for 25 seconds. This was followed by melting curve analysis at 95°C for 1 minute, and 80 cycles of 58°C for 20 seconds.

### Frequency of infected B cells

The frequency of latently infected cells in purified whole B or memory B cell populations was measured by limiting dilution analysis of the cell population followed by detection of EBV infected cells by EBV specific Real time *Taq*man DNA PCR for the W repeat sequence as described above and in [4]. This PCR can reproducibly detect a single copy of the viral genome.

### Virus shedding

Saliva sample were obtained from health volunteers. Except where noted, saliva samples were collected by mouth rinse and gargling with 5 ml of fluid. We have compared the efficiency of recovery using isotonic saline, PBS, RPMI 1640 and simple spring drinking water. We found that the recovery of viral DNA was the same regardless of the fluid used. Thereafter rinses were always performed with water and the salinity and pH adjusted by immediate addition of 10× PBS. For some experiments the saliva was clarified by centrifugation at 1500 rpm for 5 minutes at 4°C in an Allegra 6 bench top microfuge (Beckman Coulter) and/or treated with DNase (see below). Samples were stored at −80°C until all time points were collected and then analyzed simultaneously. In control experiments we found no detectable change in recovery of viral DNA after such storage periods.

100 ul of the saliva sample were digested overnight with proteinase K (PK) (Invitrogen, 0.5 mg/ml final concentration) at 55°C in a 96-well plate (8–10 replicate wells per subject per time point). 5 ul of the digested sample was used as template for EBV DNA PCR as described after deactivating the PK by heating to 95°C for 10 minutes. The amount of viral DNA in each replicate was then estimated from a standard curve generated by serial dilution (in duplicate) of an aliquot from a defined B958 culture supernatant. The amount of viral DNA in this supernatant was previously defined by limiting dilution analysis and the supernatant aliquoted and stored frozen The average value from the 8–10 replicates was then used to calculate the absolute number of virions in the 5 ml saliva sample.

### DNase treatment

DNase mix was pre-made and contained DNase (Invitrogen), 10× DNase Buffer and water. 10 ul of DNase mix was added per well of 100 ul saliva sample to a final concentration of 1 U/ul unless otherwise noted. DNase treatment was performed at room temperature for 30 minutes. DNase was deactivated by adding EDTA (2 mM) followed by incubation at 70–80°C for 30 minutes. Under these conditions virion DNA, defined as DNase resistant viral sequences, were stable for at least 1 hour whereas essentially all free cellular DNA (>99% of β-actin) was digested within 15 minutes (see Figure S3).

### Binding assay

The EBV negative Akata cell line and gastric carcinoma cell line AGS were used as target cells at 10^6^ cells per ml. AGS cells were placed in suspension by brief treatment with trypsin. Eight replicates of each binding condition were set up in a 96-well plate. Saliva samples with or without prior clarification by centrifugation and with or without DNase treatment were added into wells of target cells. The binding reaction was then performed at 37°C for 30 minutes. Cells were then pelleted by centrifugation at 1500 rpm at 4°C for 10 minutes. Cell pellets were washed 3 times with 100 ul of PBSA (0.5% BSA in PBS) per well and subsequently PK-digested at 55°C overnight. A serial dilution of B958 supernatant (EBV-producing cell line) with known numbers of virions was used as standard for the binding assay. Saliva of an EBV (-) subject and PBSA added onto target cells were used as negative controls.

### Measurement of viral burst size

For induction of lytic replication, the B95-8 B cell line was stimulated with PMA at 20 ng/ml,the Akata 2A8.1 B cell line was stimulated with a combination of PMA (20 ng/ml) and Ionomycin (1 or 10 ug/ml) [42], the AGSBX1 epithelial line was stimulated with a combination of PMA (30 ng/mL) and Butryic Acid (2.5 mM) [29] and the Hone1Akata epithelial line with PMA (40 ng/mL) and Butryic Acid (3 mM) [43]. Prior to stimulation, the cell lines were synchronized by reseeding in media overnight at 5×10^5^/ml for B cells and 3–5×106/ml for epithelial cells. B cells were harvested at 48 hours for intracellular VCA (viral capsid antigen) staining and FACS analysis and culture supernatants were harvested for EBV specific DNA PCR at 72 hours. Epithelial cells were harvested for VCA staining after 96 hours and for PCR from 96–174 hours.

For intracellular VCA staining, harvested cells were washed once with PBS and pelleted by centrifugation at 1300 rpm for 7 minutes at 4°C. Cell pellets were resuspended at 10^6^ cells per 200 ul of fixing buffer (4% formaldehyde in PBS) and incubated at room temperature for 10 minutes. Once the cells were fixed, 1 ml of PBS was added per 10^6^ cells and the cell pellets were dissociated by vortexing, and then re-pelleted by centrifugation. The PBS wash step was repeated again before resuspension into 200 ul of permeabilization buffer (8% FBS, 0.02% NaN3, 0.04% Saponin, 2% Goat Serum in PBS) per 10^6^ cells, followed by incubation at 4°C for 30 minutes. All staining was performed in FACS tubes at 10^6^ cells per tube. Cells were stained with either 200 ul of mouse IgG2a isotype control (Caltag) or 200 ul of mouse anti-VCA-IgG2a antibody (Capricorn) per 10^6^ cells diluted 1∶1000 in permeabilization buffer and incubated at 37°C for 30 minutes. The cells were then washed twice with 1 ml wash buffer (8% FBS, 0.02% NaN3, 0.04% Saponin in PBS) and pelleted. Next, cells were resuspended in 100 ul of a 1∶10,000 dilution of Alexa Fluor 488 or 647 conjugated goat anti-mouse IgG (H+L) (Molecular Probes) diluted in permeabilization buffer and incubated at 37°C for 30 min in the dark. Then, the cells were washed twice by vortexing in 2 ml of wash buffer and pelleted by centrifugation. Finally, cells were resuspended in 300 ul of FACS buffer (8% FBS, 0.02% NaN3 in PBS) for analysis on a FACS Caliber flow cytometer or 60 ul of FACS buffer for analysis on an Amnis ImageStream with IDEA analytical software to confirm the authenticity of the staining.

EBV DNA content of the culture supernatants was measured by DNA PCR with or without DNase treatment as described for the saliva samples.

### Simulations and statistical analysis

Simulations and statistical analysis (CDF and adjusted R^2^ linear regression analysis) were performed with MatLab software. To study linearity in the expected CDF for a limited number of data points with various numbers of hypothetical plaques a simulation was run. In this simulation plaques were arbitrarily assigned to undergo exponential growth. Growth of the plaques was initiated randomly and the sum of infected cells assessed at 20 random time points picked using a Monte Carlo algorithm. For details see Figure S2 and associated text.

## Supporting Information

Figure S1 Saliva virus is not depleted even after multiple large volume mouth gargles and rinses.
(0.02 MB PDF)

Figure S2 Simulated CDF's for infections involving various numbers of hypothetical plaques sampled randomly 20 times. CDFs for two simulations are shown for each number of plaques together with their best-fit linear approximation. The number of plaques simulated (n) and the resulting value for the adjusted R^2^ are given above each plot. For a detailed discussion see following text.
(0.05 MB PDF)

Figure S3 The majority of EBV DNA is DNaseresistant whereas essentially all cellular DNA is sensitive.
(0.03 MB PDF)

Figure S4 There is no correlation between the frequency of infected mBlat (FOI) in the blood and the levels of shed virus in four different types of saliva sample preparations from 5 subjects.
(0.02 MB PDF)

Figure S5 There is no correlation between the frequency of infected mBlatin the blood (FOI) and the levels of virus that binds to B cells (Akata) or epithelial cells (AGS) in two different types of saliva sample preparations from 5 subjects.
(0.03 MB PDF)

Table S1 There is no correlation between the frequency of infected mBlat (FOI) in the blood and the levels of virus shed into saliva. This data is represented graphically in Figure 5.
(0.02 MB PDF)

Text S1 Empirical CDF of shedding suggests there are at most 3 plaques at any one time.
(0.06 MB PDF)

Text S2 Why we have not observed the growth phase.
(0.04 MB PDF)
