# Supplementary material for: The Dynamics of EBV Shedding Implicate a Central Role for Epithelial Cells in Amplifying Viral Output
Source: PLoS Pathog. 2009 Jul 3;5(7):e1000496. doi: 10.1371/journal.ppat.1000496 (PMC2698984; doi:10.1371/journal.ppat.1000496)
Supplement: Figure S1 — Saliva virus is not depleted even after multiple large volume mouth gargles and rinses. (0.02 MB PDF) [file ppat.1000496.s001.pdf]

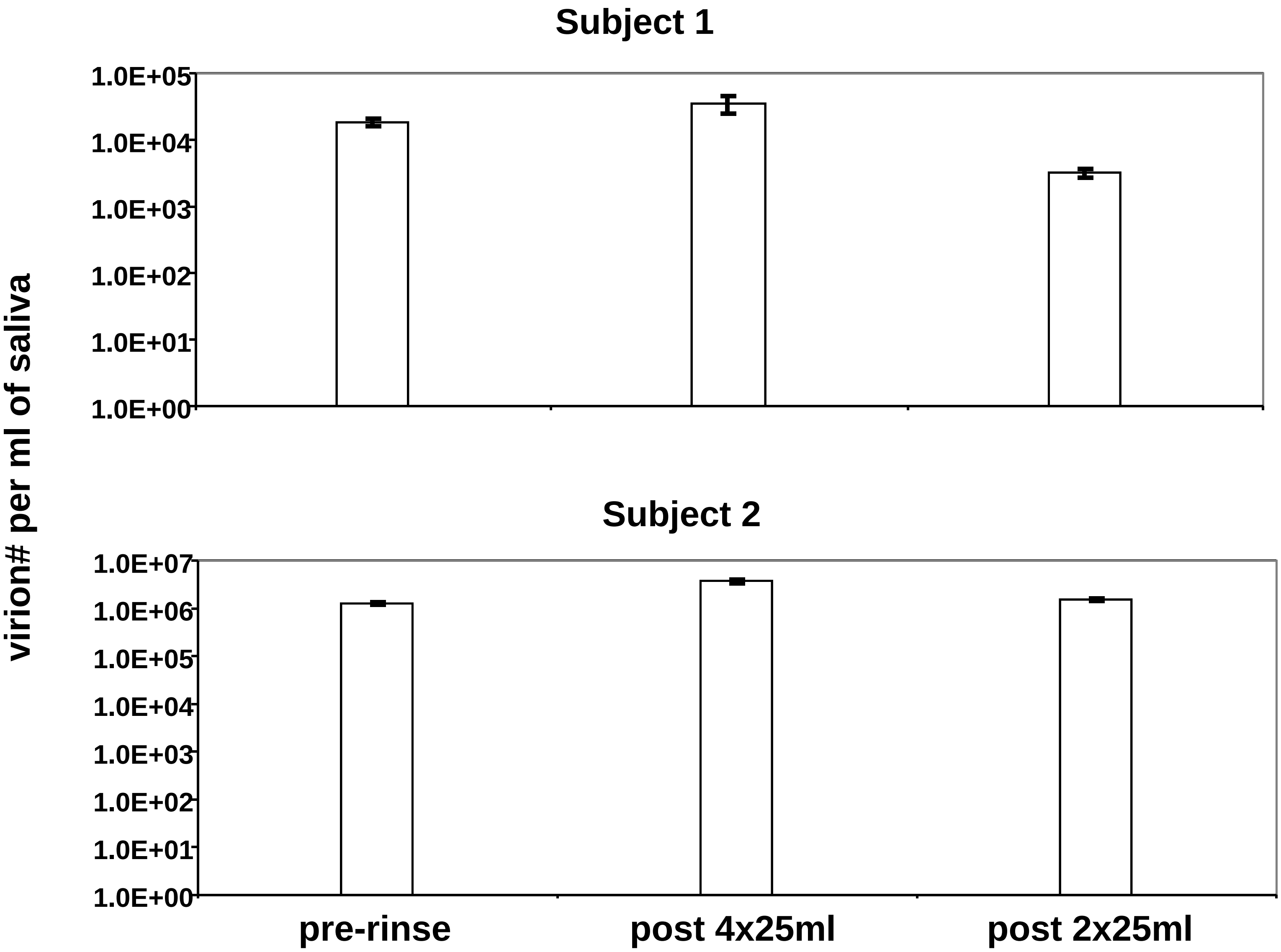

Figure S1. Saliva virus is not depleted even after multiple large volume mouth gargles and rinses.
