# Supplementary material for: The Dynamics of EBV Shedding Implicate a Central Role for Epithelial Cells in Amplifying Viral Output
Source: PLoS Pathog. 2009 Jul 3;5(7):e1000496. doi: 10.1371/journal.ppat.1000496 (PMC2698984; doi:10.1371/journal.ppat.1000496)
Supplement: Figure S2 — Simulated CDF's for infections involving various numbers of hypothetical plaques sampled randomly 20 times. CDFs for two simulations are shown for each number of plaques together with their best-fit linear approximation. The number of plaques simulated (n) and the resulting value for the adjusted R2 are given above each plot. For a detailed discussion see following text. (0.05 MB PDF) [file ppat.1000496.s002.pdf]

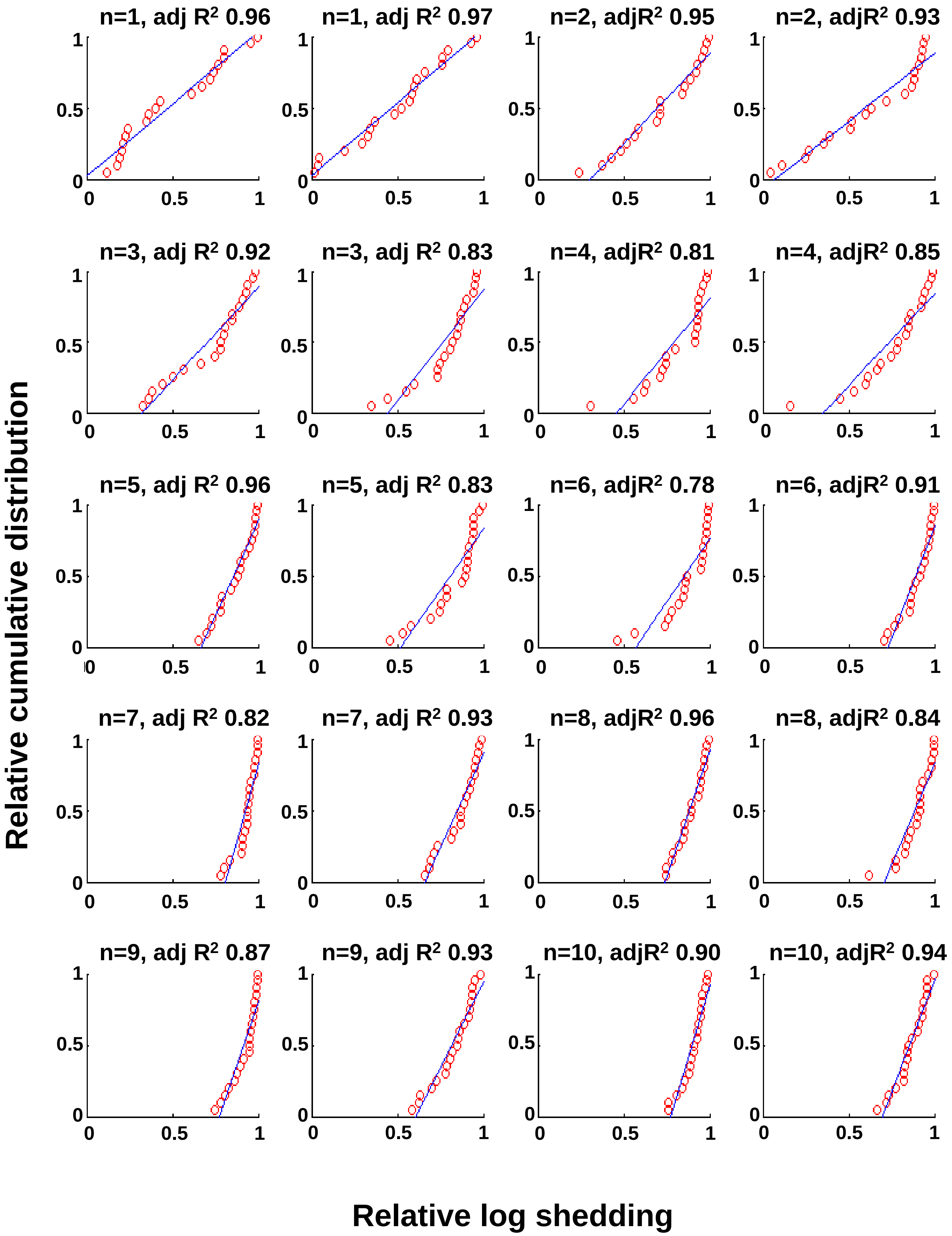
n=1, adj R^2 0.96
n=1, adj R^2 0.97
n=2, adjR^2 0.95
n=2, adjR^2 0.93
n=3, adj R^2 0.92
n=3, adj R^2 0.83
n=4, adjR^2 0.81
n=4, adjR^2 0.85
n=5, adj R^2 0.96
n=5, adj R^2 0.83
n=6, adjR^2 0.78
n=6, adjR^2 0.91
n=7, adj R^2 0.82
n=7, adj R^2 0.93
n=8, adjR^2 0.96
n=8, adjR^2 0.84
n=9, adj R^2 0.87
n=9, adj R^2 0.93
n=10, adjR^2 0.90
n=10, adjR^2 0.94
Relative cumulative distribution
Relative log shedding

Figure S2

Legend to Figure S2. Simulated CDF's for infections involving various numbers of hypothetical plaques sampled randomly 20 times. CDFs for two simulations are shown for each number of plaques together with their best-fit linear approximation. The number of plaques simulated (n) and the resulting value for the adjusted R^2 are given above each plot. For a detailed discussion see following text.
