# Supplementary material for: The Dynamics of EBV Shedding Implicate a Central Role for Epithelial Cells in Amplifying Viral Output
Source: PLoS Pathog. 2009 Jul 3;5(7):e1000496. doi: 10.1371/journal.ppat.1000496 (PMC2698984; doi:10.1371/journal.ppat.1000496)
Supplement: Figure S3 — The majority of EBV DNA is DNaseresistant whereas essentially all cellular DNA is sensitive. (0.03 MB PDF) [file ppat.1000496.s003.pdf]

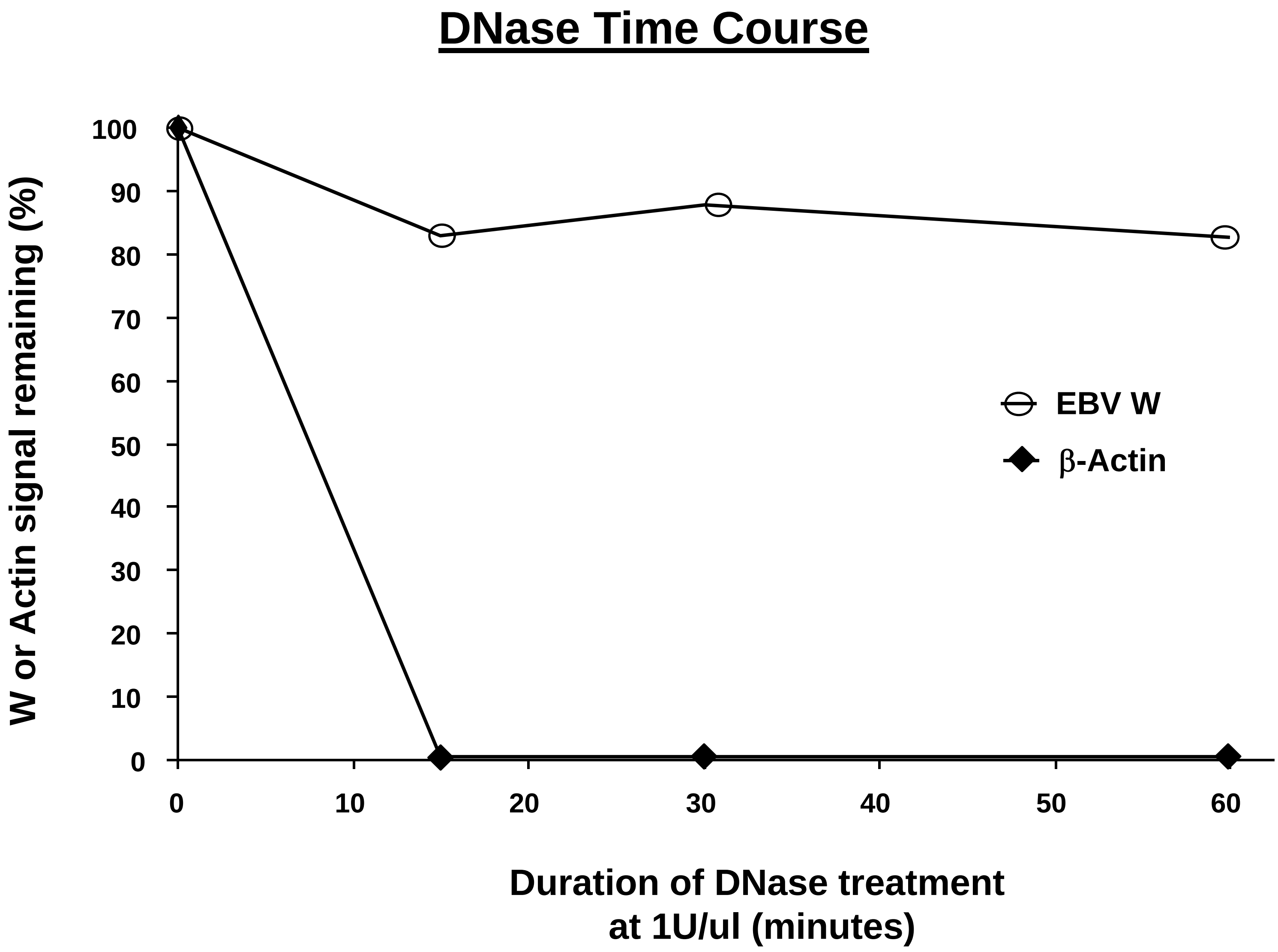

Figure S3 The majority of EBV DNA is DNase resistant whereas essentially all cellular DNA is sensitive.
