# Supplementary material for: The Dynamics of EBV Shedding Implicate a Central Role for Epithelial Cells in Amplifying Viral Output
Source: PLoS Pathog. 2009 Jul 3;5(7):e1000496. doi: 10.1371/journal.ppat.1000496 (PMC2698984; doi:10.1371/journal.ppat.1000496)
Supplement: Figure S4 — There is no correlation between the frequency of infected mBlat (FOI) in the blood and the levels of shed virus in four different types of saliva sample preparations from 5 subjects. (0.02 MB PDF) [file ppat.1000496.s004.pdf]

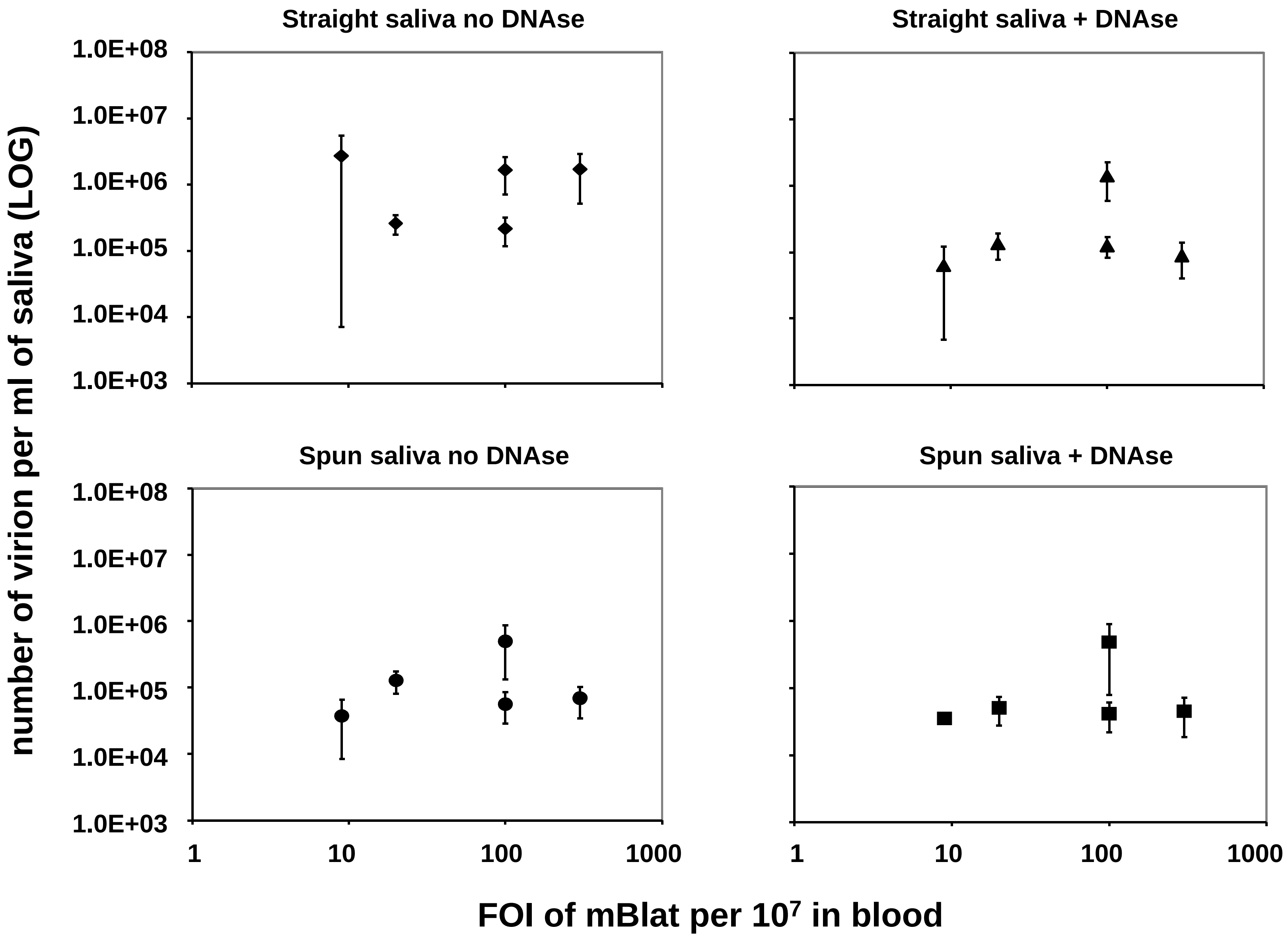

Figure S4 There is no correlation between the frequency of infected mBlat (FOI) in the blood and the levels of shed virus in four different types of saliva sample preparations from 5 subjects.
