# Supplementary material for: The Dynamics of EBV Shedding Implicate a Central Role for Epithelial Cells in Amplifying Viral Output
Source: PLoS Pathog. 2009 Jul 3;5(7):e1000496. doi: 10.1371/journal.ppat.1000496 (PMC2698984; doi:10.1371/journal.ppat.1000496)
Supplement: Figure S5 — There is no correlation between the frequency of infected mBlatin the blood (FOI) and the levels of virus that binds to B cells (Akata) or epithelial cells (AGS) in two different types of saliva sample preparations from 5 subjects. (0.03 MB PDF) [file ppat.1000496.s005.pdf]

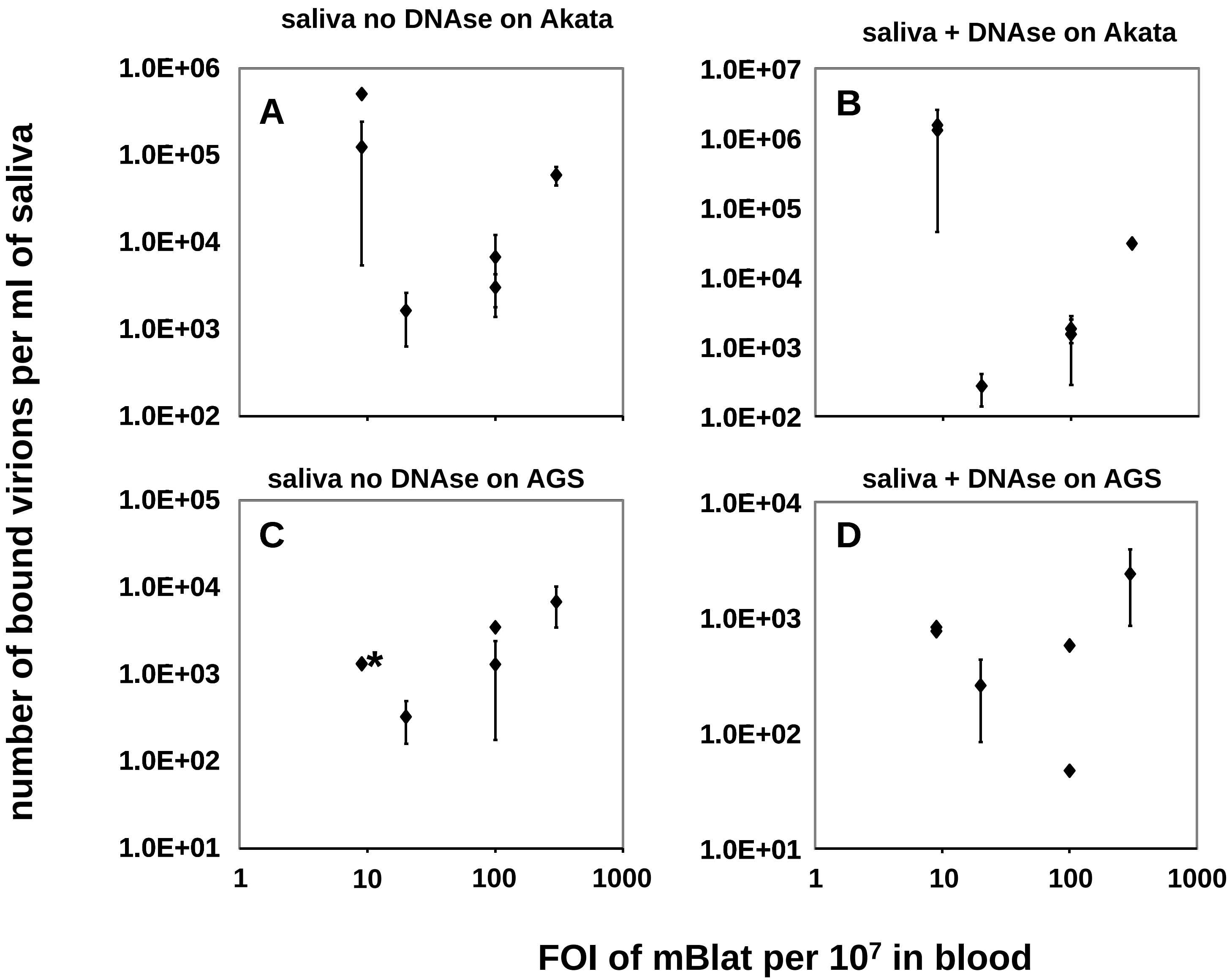

Figure S5 There is no correlation between the frequency of infected mBlat in the blood (FOI) and the levels of virus that binds to B cells (Akata) or epithelial cells (AGS) in two different types of saliva sample preparations from 5 subjects.
