# Supplementary material for: The Dynamics of EBV Shedding Implicate a Central Role for Epithelial Cells in Amplifying Viral Output
Source: PLoS Pathog. 2009 Jul 3;5(7):e1000496. doi: 10.1371/journal.ppat.1000496 (PMC2698984; doi:10.1371/journal.ppat.1000496)
Supplement: Table S1 — There is no correlation between the frequency of infected mBlat (FOI) in the blood and the levels of virus shed into saliva. This data is represented graphically in Figure 5. (0.02 MB PDF) [file ppat.1000496.s006.pdf]

Table S1: There is no correlation between the frequency of infected mBlat (FOI) in the blood and the levels of virus shed into saliva.

Subject	blood FOI	Mean shedding/ml	Median shedding/ml	SEM
8	300	4.25E+05	1.18E+04	2.36E+05
5	100	4.12E+05	1.97E+04	1.77E+05
4	100	3.67E+04	2.91E+03	2.82E+04
7	100	1.15E+06	6.41E+04	5.53E+05
6	40	3.18E+04	6.78E+03	1.52E+04
3	30	4.95E+06	1.76E+06	1.36E+06
2	20	2.16E+05	2.39E+03	7.30E+04
1	9	4.55E+05	1.50E+05	4.42E+05

This data is represented graphically in Figure 5.
