# Supplementary material for: The Dynamics of EBV Shedding Implicate a Central Role for Epithelial Cells in Amplifying Viral Output
Source: PLoS Pathog. 2009 Jul 3;5(7):e1000496. doi: 10.1371/journal.ppat.1000496 (PMC2698984; doi:10.1371/journal.ppat.1000496)
Supplement: Text S1 — Empirical CDF of shedding suggests there are at most 3 plaques at any one time. (0.06 MB PDF) [file ppat.1000496.s007.pdf]

Empirical CDF of shedding suggests there are at most 3 plaques at any one time.

We have seen that the empirical cumulative distribtion function(CDF) for the log of shedding for each of our subjects is linear. We will argue here that this implies that there are only a small number of plaques at any point in time. In brief, we argue as follows: A single instance of exponential growth observed at a a random point in that growth produces a linear CDF for the log of the size of that process (here shedding). This is consistent with the observed linear CDFs. We will see that small numbers of plaques are also consistent with the observed CDFs.

A linear CDF is also consistent with a process which undergoes exponential growth for a random length of time, stabilizes and then is sampled during its constant phase. In this interpretation the empirical linear CDF depends on our not seeing the growth phase. We will see in Supplement Text S2 that small numbers of plaques could act to hide the growth phase.

Suppose that the growth of a single plaque is exponential and that this is sampled with a uniform distribution. The log of the resulting sample is uniformly distributed. Put differently, the log of this value has a linear cumulative distribution function (CDF). After normalization, if X is the random variable representing the $\log_{10}$ of shedding, its CDF has the form

$$f_X(x) = p(X \leq x) = \begin{cases} 0 & \text{if } x \leq 0 \\ x & \text{if } 0 \leq x \leq 1 \\ 1 & \text{if } 1 \leq x \end{cases}$$

Suppose now that X_1 and X_2 are random variables for the log shedding of two different plaques. Suppose that these are each distributed as above and are independent. If Y is the random variable giving the log of total shedding, we have

$$\begin{aligned} Y &= \log(10^{X_1} + 10^{X_2}) \\ &\leq \log(2\max(10^{X_1}, 10^{X_2}) \\ &= \max(X_1, X_2) + \log(2) \end{aligned}$$

Since the shedding values in question are on the order of 10 to 10^7, we will use the approximation $Y = \max(X_1, X_2)$. For $0 \leq x \leq 1$ the CDF then has the form

$$\begin{aligned} f_Y(x) &= p(Y \leq x) \\ &= p(\max(X_1, X_2) \leq x) \\ &= p(X_1 \leq x \text{ and } X_2 \leq x) \\ &= p(X_1 \leq x)p(X_2 \leq x) \\ &= x^2 \end{aligned}$$

An induction shows that if there are n plaques whose shedding is independent and distributed as above, the CDF for the log of total shedding is

$$f_n(x) = \begin{cases} 0 & \text{if } x \leq 0 \\ x^n & \text{if } 0 \leq x \leq 1 \\ 1 & \text{if } 1 \leq x \end{cases}$$

It is clear that the near-linear empirical CDFs shown in Figure 6 are consistent with our model here for a single plaque. The question arises: Is a linear empirical CDF consistent with this model for $n > 1$?

With large numbers of sample points, the empirical CDF closely approximates the CDF. With smaller numbers of sample points, a non-linear CDF can easily produce a near-linear empirical CDF in the following manner. The CDF $f_n(x) = x^n$ looks much like a line for a large portion of the region $0 \leq y = f_n(x) \leq 1$. Since the y-axis represents probability here, with some probability, a randomly chosen set of 20 points will fail to detect the CDF's deviation from linearity for $n > 1$.

For each number n of simulated plaques, $1 \leq n \leq 10$, we performed a Monte Carlo simulation to determine how often we should expect to see a linear CDF when drawing $N = 20$ points randomly from each of the above distributions. For each sample of 20 points, we computed the adjusted R^2 of the least-squares linear fit to the sample CDF. Examples of these are shown in Supplement Figure B. For each n we performed this simulation 100 times. Figure 7 shows the resulting CDF for adjusted R^2 at each value of n.

This allows us to compute the p-value for various choices of null hypothesis. For example, having chosen n, we imagine that all subject data was drawn from the distribution for n plaques. We may then enquire as to the probability of

1. Chosing 8 samples each of twenty points each of which has adjusted R^2 greater than or equal to the least observed adjusted R^2 in the subject data.

2. Chosing 8 samples each of twenty points such that the highest four adjusted R^2 values are at least as large as the median of the adjusted R^2 for the subject data.

3. Choosing 8 samples of twenty points such that seven of these have adjusted R^2 at least as large as that of the second lowest observed R^2 for the subject data.

We use $f_{\min}(n)$, $f_{\text{median}}(n)$ and $f_{7/8}(n)$ to denote the fraction of simulations for n plaques required in these three null hypostheses. We can then compute

corresponding p-values as

$$p_{\text{min}}(n) = f_{\text{min}}(n)^8$$

$$p_{\text{median}}(n) = \sum_{i=4}^{8} \binom{8}{i} f_{\text{median}}(n)^i (1 - f_{\text{median}}(n))^{8-i}$$

$$p_{7/8}(n) = \sum_{i=7}^{8} \binom{8}{i} f_{7/8}(n)^i (1 - f_{7/8}(n))^{8-i}$$

Plaque numbers that gave $p < 0.005$ for each method are given in the text.

Note that we have considered here null hypotheses where the number of plaques is fixed. For example, $p_{\text{min}}(5)$ is the probability that all adjusted R^2 are at least as good as the lowest observed values if there are always 5 plaques. One could also ask what this p-value is if there are always 5 or more plaques. In order to make this question precise, we must assign specific probabilities to the different possible plaque numbers, $n = 5, 6, \ldots$. However, $f_{\text{min}}(n)$ et al., are nearly monotone, i.e., increasing n produces little or increase in $f_{\text{min}}(n)$. As a consequence, allowing mixed numbers of plaques produces little or no increase in the resulting p-values.
