# Supplementary material for: The Dynamics of EBV Shedding Implicate a Central Role for Epithelial Cells in Amplifying Viral Output
Source: PLoS Pathog. 2009 Jul 3;5(7):e1000496. doi: 10.1371/journal.ppat.1000496 (PMC2698984; doi:10.1371/journal.ppat.1000496)
Supplement: Text S2 — Why we have not observed the growth phase. (0.04 MB PDF) [file ppat.1000496.s008.pdf]

Why we have not observed the growth phase.

We have argued that our linear empircal CDFs are the result of a process of exponential growth. This requires that we explain why we do not detect this exponential growth when shedding is measured over short intervals like hours or days. It is for this reason that we have turned to the idea that plaques grow exponentially for a random amount of time and then stablize in time. One reason for failure to observe the growth phase would be if it is short compared to the stable phase. We argue here that the presence of multiple plaques would also act to mask the growth phase. Suppose that a second plaque grows while a previous plaque is stable. If the first plaque is larger than the second, it will hide the entire growth phase. If, on the other hand, the first plaque has log shedding half that of the second, it will still mask the first half of the second plaque's growth phase. Let us normalize the range of log shedding to the interval from 0 to 1, and denote the log shedding of the first and second plaques by x and y. If $x > y$, the first plaque will mask the entire growth phase of the second. If $x < y$, the fraction of the second plaque's growth phase that will be hidden is $\frac{x}{y}$. The fraction that is hidden is thus $\min(1, \frac{x}{y})$. To find the expected value for this, we integrate over the range of these two variables giving

$$\frac{3}{4} = \int_0^1 \int_0^1 \min\left(1, \frac{x}{y}\right)\ dx\ dy.$$

Since shedding is (nearly) continuous, each plaque is subject to this kind of masking when it first appears. Thus on average, we should see the growth phase at most a quarter of the time it is present. If the growth phase is equal in duration to the stable phase, the growth phase is present only half the time, and thus, should be observed in only an eighth of all attempts to measure it.

Exactly how much masking is provided by multiple plaques depends both on the number of plaques and the relative lengths of the growth phase and the stable phase. While the exact degree of masking is uncertain, it is very likely high enough to explain our failure to observe the growth phase in the limited number of instances where we have taken measurements over closely spaced time points.
